# Neuromuscular Activity during Cycling Performance in Hot/Dry and Hot/Humid Conditions

**DOI:** 10.3390/life11111149

**Published:** 2021-10-28

**Authors:** Michelle Baillot, Olivier Hue, Trong Than Tran, Sophie Antoine-Jonville

**Affiliations:** 1 Laboratoire ACTES, UPRES-EA 3596 UFR-STAPS, Université des Antilles, 97157 Pointe à Pitre, Guadeloupe, France; michellemanip@gmail.com (M.B.); sophie.antoine@univ-avignon.fr (S.A.-J.); 2 Department of Physical Education, University of Economics-Technology for Industries, Hai Ba Trung, Hanoi 11019, Vietnam; ttthan.uneti@moet.edu.vn; 3 Laboratoire de Pharm-Ecologie Cardiorespiratoire (LAPEC EA4278), Avignon Université, 84000 Avignon, France

**Keywords:** thermoregulation, heat loss, hot/humid climate, electromyogram, aerobic exercise

## Abstract

To determine the relationships between limiting factors and neuromuscular activity during a self-paced 20-km cycling time trial and evaluate the effect of environmental conditions on fatigue indices. Methods: Ten endurance-trained and heat-acclimated athletes performed in three conditions (ambient temperature, relative humidity): HUMID (30 °C, 90%), DRY (35 °C, 46%) and NEUTRAL (22 °C, 55%). Voluntary muscular contractions and electromagnetic stimulations were recorded before and after the time trials to assess fatigue. The data on performance, temperature, heat storage, electromyogram, heart rate and rating of perceived exertion data were analyzed. Results: Performance was impaired in DRY and HUMID compared with NEUTRAL environment (*p* < 0.05). The force developed by the vastus lateral muscle during stimulation of the femoral nerve remained unchanged across conditions. The percentage of integrated electromyogram activity, normalized by the value attained during the pre-trial maximal voluntary contraction, decreased significantly throughout the trial only in HUMID condition (*p* < 0.01). Neuromuscular activity in peripheral skeletal muscle started to fall from the 11th km in HUMID and the 15th km in DRY condition, although core temperature did not reach critical values. Conclusions: These alterations suggest that afferences from core/skin temperature regulate the central neural motor drive, reducing the active muscle recruited during prolonged exercise in the heat in order to prevent the system from hyperthermia.

## 1. Introduction

The effects of environmental temperature on heat tolerance during prolonged exercise has been studied over the past decade, and the results indicate that hyperthermia contributes to voluntary exhaustion [1]. This altered performance in hot climate has two possible explanations: first, above a critical core temperature, homeostatic regulation of several physiological functions is impaired [2] and, second, input from thermal receptors can lead to changes in the central nervous system (CNS) that decrease the intensity of exercise performance in order to reduce heat storage in the body [3]. During high-intensity exercise, lower cardiovascular function may be an important factor as it can reduce muscular oxygen delivery [4]. The CNS hypothesis seems to be relevant mainly during prolonged moderate-intensity exercise and when the capacity for evaporative heat release is hampered [5]. In hot/dry conditions, heat loss mainly occurs through sweat evaporation. In a tropical climate (i.e., hot/humid environment), this mechanism is limited due to the high percentage of humidity. Therefore, hyperthermia and thus voluntary exhaustion will limit exercise capacity [6]. From a practical point of view, the understanding of the limiting factors to performance in warm humid environment is of interest for the development of optimal heatstroke prevention strategies while preserving performance.

We examined the first hypothesis—that is, that with the failure in homeostasis above a critical core temperature, peripheral factors are the main factors to explain exhaustion—and tested whether or not “central fatigue” causes impaired aerobic performance in hot/humid conditions. It has been demonstrated that it is not ambient temperature, but rather vapor pressure that determines self-paced performance [7]. Tucker et al. indeed showed that power output during self-paced exercise in the heat was lower, whereas heart rate, rating of perceived exertion (RPE) and core temperature were similar to those observed in cool conditions [8]. We demonstrated earlier that tropical climate impaired aerobic performance in ecological conditions. We thus found it interesting to evaluate whether hot/humid conditions would be more stressful than hot/dry conditions on physiological functions [9,10], as compared with a control condition.

Our studies tried to evaluate the impact of the tropical climate on aerobic performance. Thus, we conducted an experiment where we could compare hot/dry to hot/humid conditions. As the dry heat has been well documented [8], we used the same protocol to highlight the more significant impact of hot/humid conditions on aerobic performance. To do so, we conducted a study on acclimated athletes performing a self-paced cycling time trial over 20 km in three laboratory conditions (neutral, hot/dry and hot/humid). The previous studies cited emphasized the detrimental consequences of the tropical climate in outdoor conditions, showing that the athletes did not reach critical Trec even when they took part in a competition (half ironman and 27 km running trail).

We hypothesized that free-intensity cycling exercise in a tropical climate would be influenced by tropical specificities (i.e., the impossibility of reducing core temperature through evaporation and high fluid loss), but that performance would be reduced as a way to prevent a critical core temperature from being reached.

## 2. Materials and Methods

### 2.1. Population

Ten heat-acclimated trained male cyclists and triathletes (VO2max > 55 mL·min^−1^·kg^−1^) were recruited for the study. Participants were naturally acclimatized to tropical climate as they had all been living in Guadeloupe for more than 2 years. They were acclimatized to outdoor exercise (>10 h/week) at the time of this research. They were informed of the associated risks with it and provided informed written consent before participating. The University Ethics Committee approved the study, wich was carried out in line with the Declaration of Helsinki. The participants’ mean (±SD) age, height, body mass, BMI, maximal heart rate (HR), maximal oxygen uptake (VO2max) and peak power output at VO2max were 36.0 ± 9.3 y, 177 ± 5 cm, 70.8 ± 8.3 kg, 22.6 kg/m^2^, 177 ± 10 bpm, 61.8 ± 6.3 mL·min^−1^·kg^−1^ and 290 ± 59 W, respectively.

### 2.2. Preliminary Testing

The participants came to our laboratory for anthropometric measurements and preliminary testing. Height and body mass measurements were made with a high-precision stadiometer and balance (Model 770, Seca, Bonn, Germany). The participants then used a cycle ergometer (Monark Weight Ergometer 814 E, Varberg, Sweden) for an incremental exercise test to evaluate their VO2max. Before beginning the test, they started with a 10 min self-paced warm-up at an initial power output of 3 W/kg body mass. We increased the workload by 30 W/min until they reached exhaustion. Gas exchange (ZAN Ferraris, Cardiorespiratory System, Oberthulba, Germany) was assessed over the entire test. The test was finished when the subject was unable to match the required power output, VO2max showed no increase as intensity increased, the respiratory exchange ratio was > 1.05, HR remained within 10 bpm of the age-predicted maximum of 220− age, or participants could not remain in a seated position

### 2.3. Trial for Familiarization

Within 7 days of VO2max determination, the participants returned to the laboratory to familiarize themselves with the Borg scale, the equipment and the laboratory conditions. We considered that even though the participants were regularly trained and the session presentation order was randomized, each experimental session could represent a specific impact, whether from the repetition of maximal isometric contractions or the « time trial » format. In order to prevent from abnormal accumulation of fatigue within the training plan, this decision was taken by the researchers, aware that it was potentially unnecessary but that this standardization had more interests than risks.

In this trial, they completed a 20-km cycling time trial on an ergometer (Travel Elite “fluid” model, CT 01, Fontaniva, Italy) that let them ride their own bicycles in a seated position, at an ambient temperature of 22.0 ± 0.5 °C, 60 ± 6% relative humidity (RH), under ad libitum conditions. They were requested to “ride as fast as possible” and were not given any feedback other than their elapsed distance at the completion of each kilometer. All procedures and conditions were exactly the same as those of the following experimental trials.

### 2.4. Design

Following a random crossover design, each subject completed three experimental 20-km cycling time trials separated by 7 days. Methodology: They performed these randomized time trials at 29.7 ± 0.5 °C, 90.4 ± 6.5% RH (HUMID), at 32.8 ± 1.6 °C, 46.2 ± 5.5% RH (DRY), and at 23.2 ± 0.7 °C, 56.3 ± 3.3% RH (NEUTRAL) with vapor pressure 4.2 kPa (HUMID), 5.6 kPa (DRY), and 2.6 kPa NEUTRAL.

They were asked to refrain from alcohol and exercise for 24 h before the experimental trials. Before starting the trial days, they ingested a standard breakfast that comprised food and 600 mL of a beverage, but no tea or coffee. The trials for all participants were set for 11 AM to reduce the effects of circadian variation. During the trials, they wore the same cycling clothes and a chest-strapped heart rate monitor.

### 2.5. Twitch Force of the Vastus Lateralis Muscle

A widely accepted painless and supramaximal method of nerve stimulation was used to assess quadriceps strength and fatigue [11,12]. The femoral nerve was stimulated using a magnetic stimulator (Inomed, Magstim 200, Whitland, Wales, UK) and a 70-mm figure-8 coil. A set of five potential twitches at 100% stimulator output was performed before and after the exercise. The mean of the three highest contractions was used to evaluate the twitch force of the vastus lateralis muscle (TWq).

### 2.6. Testing for Maximal Voluntary Contraction Testing

Before all trials and immediately after the TWq measurements, the strength of the vastus lateralis muscle was measured. The right leg was fastened to the dynamometer above the lateral malleolus. The subject’s right knee extensor strength was calculated and the electromyogram (EMG) activity of the vastus lateralis muscle was evaluated. Each subject performed three submaximal preliminary contractions before performing three brief maximal contractions (2 s), each separated by 2 min of rest. Each participant was verbally stimulated with encouragement specifically to produce maximal force during each contraction. At the end of each try, the subject was made aware of the force reached and whether a 1 s-plateau had been attained. We used the highest force to determine maximal voluntary contractions (MVC) and normalize the EMG signal obtained in the 20-km time trial.

### 2.7. EMG Testing

**Figure 1:**
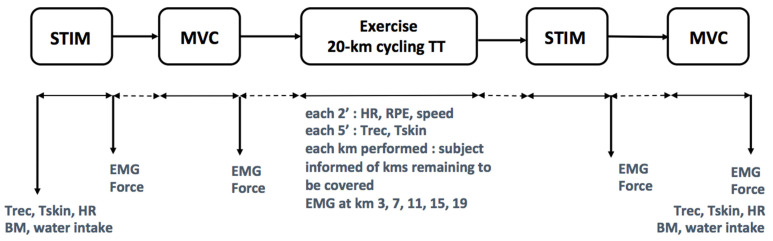
Schematic representation of the study design. Trec: rectal temperature; Tskin: skin temperature; HR: heart rate; EMG: electromyogram activity of the vastus lateralis muscle; RPE: rating of perceived exertion; STIM: magnetic stimulations; MVC: maximal voluntary contractions.

During each MVC and following the 20-km time trial, the EMG activity of the vastus lateralis muscle was recorded (Figure 1). Once the skin was shaved and cleaned with 95% ethanol, in conformity with methods formerly defined [13], the electrodes (3M Health Care, model 2223, Neuss, Germany) were positioned over the muscle belly of the vastus lateralis and coupled to a pre-amplifier. The electrodes were secured to the skin using tape, and a bandage (Elastoplast, France) was draped around the electrode to reduce sweat interference. EMG signals were captured at 2500 Hz during the MVCs and the 20-km time trials. During the time trials, EMG activity during the trials was recorded at 3, 7, 11, 15, 19 km. For signal analysis, 2.5 s of data were analyzed as the participants had chosen their own cycling cadence. The raw EMG signals were full-wave rectified, with a high-pass filter removing the movement artefacts and the signals were then smoothed with a low-pass filter. This was performed using BIOPAC (MP 30 System, Santa Barbara, CA, USA) and AcqKnowledge software (3.2 acquisition software, Biopac Systems, Santa Barbara, CA, USA). This iEMG was used for following analysis. All EMG data were normalized by dividing the EMG value obtained at 3, 7, 11, 15, 19 km by the EMG value during the MVC performed before the start of each time trial. iEMG was thus given as a percentage of this MVC data. This process of iEMG normalization was shown to be consistent and effective in cycling trials [14]. Therefore, using this methodology, the neuromuscular responses (iEMG) in self-paced cycling in the heat are reproducible between trials using this methodology in warm and humid conditions [15].

### 2.8. Temperatures

Following MVC testing, participants introduced a rectal thermometer (YSI409AC, Yellow Springs Instruments, Yellow Springs, OH, USA) 10 cm beyond the anal sphincter. Saltin and Hermansen have demonstrated that rectal temperature is practically identical to esophageal temperature when cycling at elevated work rates [16]. Thermocouples (YSI 427, OH, USA) were firmly fastened to the sternum region, forearm, and both the left mid-thigh and calf in order to calculate skin temperature (Tskin) and the total body temperature. The heat storage was calculated using the equation Qs = Qc,T1-Qc,T2 where Qs is heat storage in kJ, Qc,T1 is heat content at time 1 and Qc,T2 is the heat content at time 2 [17]. Participants then performed a self-paced, 2-min warm-up to the same initial values for HR, rate of perceived exertion (RPE) and rectal temperature (Trec) in the three trials. At the end of the warm-up, Tskin, Trec and HR were recorded. A telethermometer, accurate to 0.1 °C (YSI 400 series) was used to measure temperatures every 5 min.

At the start of the trial, HR and RPE were recorded and monitored every 2 min using respectively a Polar RX-875 heart rate monitor (Polar Electro, Kempele, Finland) and the Borg category ratio scale [18]. Nude body mass was recorded before and after the trial after sweat was toweled off. Water consumption during the trial was measured (in L). Weight loss rate (BML in kg·h^−1^) was calculated by the change in body mass adjusted for fluid consumption. This weight loss reflected sweat rate, but was not adjusted for other body weight losses caused by irreversible fuel oxidation, since it was estimated to be basically similar in the three conditions.

### 2.9. Statistical Analysis

MVC, EMG data, speed, temperatures, heat storage rate, RPE and HR data were analyzed using a two-way ANOVA for repeated measures to determine the temperature and time interactions. Rate of weight loss and fluid ingestion were analyzed using a one-way ANOVA for repeated measures. When a significant effect was observed, post-hoc comparisons were made using Tukey’s “honestly significantly different” (HSD) test for pairwise comparisons. Performance times were investigated operating a paired *t*-test. Significance was accepted for all analyses at *p* ≤ 0.05. Data are presented as means ± SEM.

## 3. Results

### Performance

**Figure 2:**
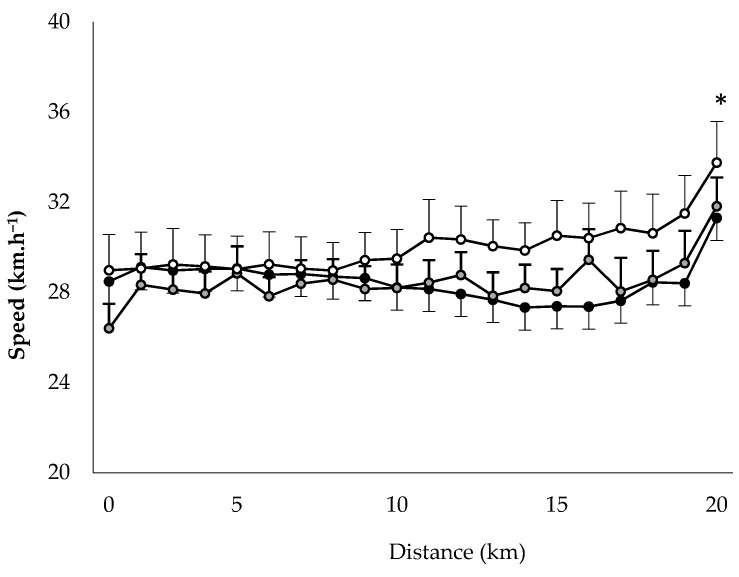
Speed during the 20-km tests in the three conditions HUMID (black), DRY (gray) and NEUTRAL (white). * Significantly greater than in the previous intervals in the three conditions.

The times for the 20-km time trial were 44.6 ± 2.5, 43.9 ± 1.7 and 40.7 ± 1.8 min in HUMID, DRY and NEUTRAL, respectively. Repeated measures ANOVA revealed a significant main effect of Condition (*p* = 0.014, ηp2 = 0.65) on the times and they were significantly greater in HUMID (*p* = 0.019) and DRY (*p* = 0.009) than in NEUTRAL. Mean speed expressed in km·h^−1^ was 28.5 ± 4.2, 28.5 ± 3.5 and 30.0 ± 4.8 in HUMID, DRY and NEUTRAL, respectively (*p* = 0.021). In the three conditions, the speed in the last 10% interval was significantly higher than that in the previous intervals (Figure 2).

**Figure 3:**
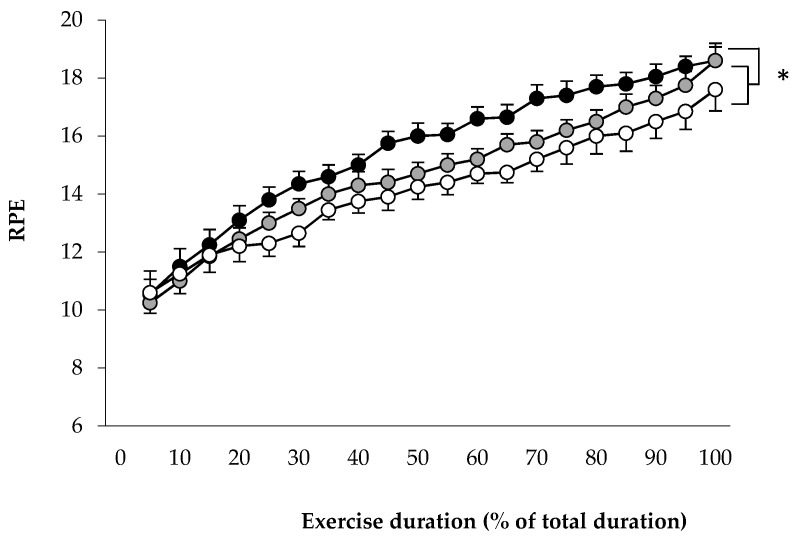
Rating of perceived exertion during the three conditions HUMID (black), DRY (gray) and NEUTRAL (white). * Significantly different from neutral (*p* ≤ 0.05).

Repeated measures ANOVA revealed a significant main effect of Period (*p* = 0.029, ηp2 = 0.58) on RPE. RPE was significantly greater in HUMID compared with DRY (*p* = 0.025) and NEUTRAL (*p* = 0.006) (Figure 3).

**Figure 4:**
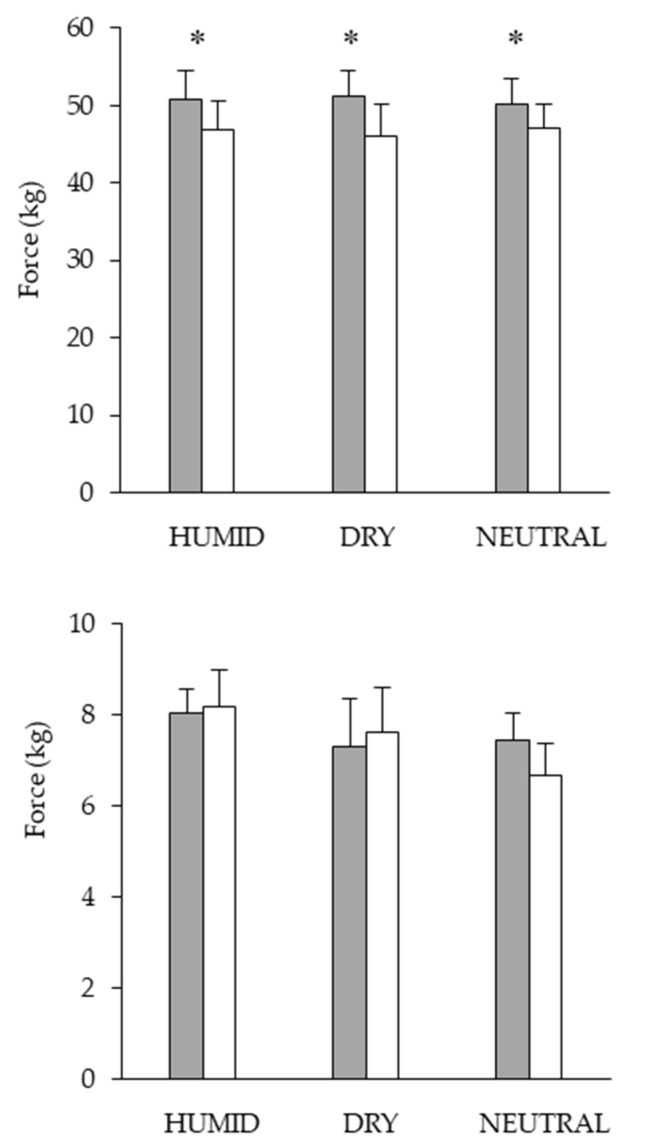
Force developed during maximal voluntary contractions (**upper figure**) and during magnetic stimulations (**lower figure**) from the quadriceps pre-trial (gray) and post-trial (white) in HUMID (30 °C), DRY (35 °C) and NEUTRAL (22 °C) conditions. Means ± SEM for 10 participants. * Significantly different from POST (*p* ≤ 0.05).

The maximal voluntary contractions of the quadriceps were affected by exercise, showing the same amplitude in the three conditions (*p* = 0.002, ηp2 = 0.66) (Figure 4). No statistically significant effect of Condition or effect of Condition × Period was found for the force developed by the vastus lateralis muscle during the magnetic stimulations (Figure 4).

**Figure 5:**
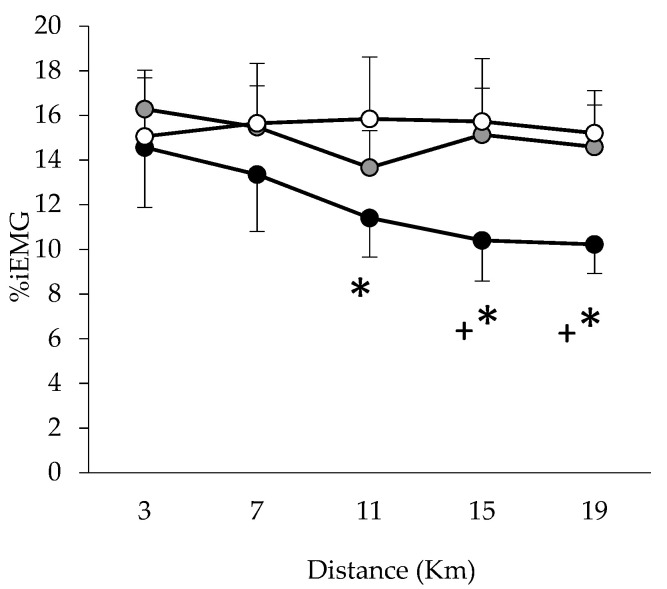
Integrated electromyogram from the vastus lateralis muscle at 3, 7, 11, 15 and 19 km during trials in the three conditions HUMID (black), DRY (gray) and NEUTRAL (white). Means ± SEM for 10 participants. * Significantly different from NEUTRAL (*p* ≤ 0.05) + Significantly different from DRY (*p* ≤ 0.05). %iEMG: Percentage of integrated electromyography.

Repeated measures ANOVA revealed a significant main effect of Condition (*p* = 0.036, ηp2 = 0.56) and the Condition × Period interaction (*p* = 0.047, ηp2 = 0.98) for %iEMG. The %iEMG activity was not different at the start of the trials, and no significant differences were found during the first 7 km of the trials between conditions, but %iEMG in HUMID was lower than in NEUTRAL at 11, 15 and 19 km, and lower than in DRY at 15 and 19 km (Figure 5).

**Figure 6:**
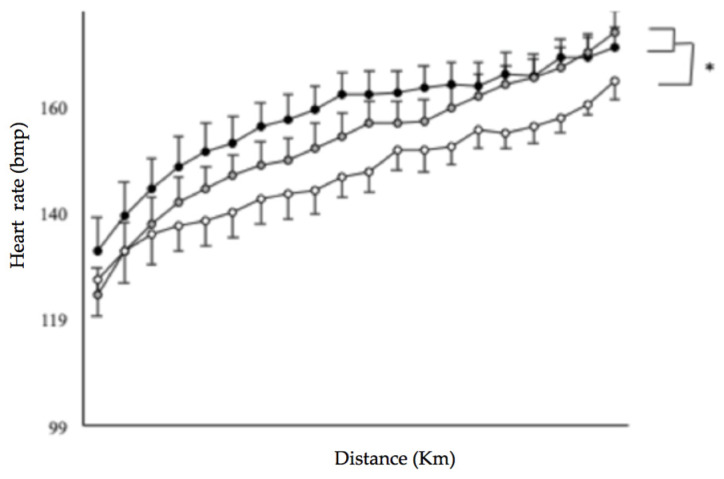
HR during the 20-km tests in the three conditions HUMID (black), DRY (gray) and NEUTRAL (white). * Significantly different from NEUTRAL (*p* ≤ 0.05).

Repeated measures ANOVA revealed a significant main effect of Period (*p* = 0.016, ηp2 = 0.64) for HR. HR was significantly greater in HUMID (*p* = 0.003) and DRY (*p* = 0.050) than in NEUTRAL (Figure 6).

**Figure 7:**
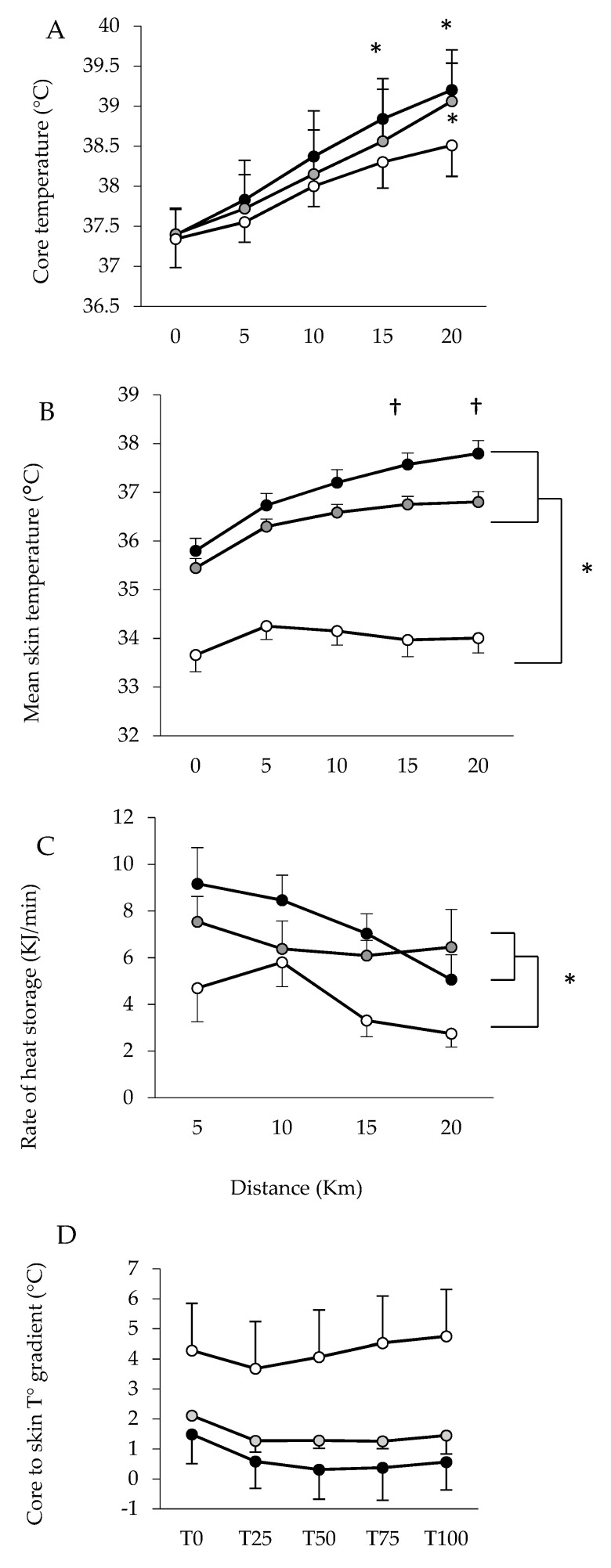
Core temperature (**A**), mean skin temperature (**B**), rate of body heat storage (**C**), core to skin gradient (**D**) in the three conditions HUMID (black), DRY (gray) and NEUTRAL (white). * Significantly different from NEUTRAL. † Significantly different from DRY.

Figure 7 shows the change in rectal temperature (A), skin temperature (B), the rate of heat storage (C) and the core to skin gradient (D) throughout the experiments. Repeated measures ANOVA revealed a significant main effect of Period (*p* < 0.0001, ηp2 = 0.94), Condition (*p* = 0.015, ηp2 = 0.37) and the Condition × Period interaction (*p* = 0.046, ηp2 = 0.27) for rectal temperatures. Significantly higher rectal temperatures were found in HUMID compared with NEUTRAL at 15 and 20 km, and in DRY compared with NEUTRAL at 20 km. Repeated measures ANOVA revealed a significant main effect of Period (*p* < 0.001, ηp2 = 0.67), Condition (*p* < 0.001, ηp2 = 0.88) and the Condition × Period interaction (*p* < 0.001, ηp2 = 0.46) for skin temperatures. Significantly higher mean Tskin was found in HUMID compared with DRY at 15 and 20 km, and in HUMID and DRY compared with NEUTRAL throughout the trial. Repeated measures ANOVA revealed a significant main effect of Condition (*p* = 0.031, ηp2 = 0.57) for heat storage. The rate of heat storage was significantly greater in HUMID (*p* = 0.008) and DRY (*p* = 0.018) compared with NEUTRAL.

The rate of BML in kg·h^−1^ was 2.6 ± 0.5, 2.5 ± 0.9 and 1.7 ± 0.5 in HUMID, DRY and NEUTRAL, respectively. Repeated measures ANOVA revealed a significant main effect of Condition (*p* = 0.008, ηp2 = 0.70) on the rate of BML and it was significantly higher in HUMID (*p* = 0.001) than in NEUTRAL. The rate of water intake in L·h^−1^ was 1.1 ± 0.6, 1.3 ± 1.2, 0.5 ± 0.3 in HUMID, DRY and NEUTRAL, respectively. Repeated measures ANOVA revealed a significant main effect of Condition (*p* = 0.030, ηp2 = 0.58) on the rate of water intake and it was significantly higher in HUMID (*p* = 0.009) and DRY (*p* = 0.008) than in NEUTRAL.

## 4. Discussion

It has been demonstrated that the peripheral fatigue developed during exercise in the heat is not a direct cause of decreased aerobic performance, which might instead be due to “central fatigue” [4]. In hot/humid climate, aerobic performance is also impaired because of the high RH of the air. Interestingly, in this study the participants declared a significantly greater RPE, which is a subjective parameter, in HUMID compared with DRY and NEUTRAL, reflecting the strain of the environment. The force produced in the vastus lateralis muscle during the MVCs was affected by exercise to the same amplitude in the three conditions. Nevertheless, we found that TWq remained the same in NEUTRAL, DRY and HUMID, indicating that the capacity of the skeletal muscle to produce force was not altered. Thus, %iEMG significantly decreased during exercise only in the HUMID condition. The %iEMG was reduced from the 15th km in HUMID compared with DRY and from the 11th km in HUMID compared with NEUTRAL. It has been shown that a hot ambient temperature causes a reduction in iEMG [8], whereas our study demonstrated that ambient humidity was responsible for the reduction in iEMG. This result is the major novelty of this study. According to the literature, a 2 °C difference impairs perceptual responses (without impact on performance) [19], whereas in our study, even a 1 °C difference in Tskin was associated with lower iEMG (but not performance). Moreover, the core to skin temperature gradient was enhanced in NEUTRAL compared with DRY and HUMID, indicating that convective heat loss was facilitated in NEUTRAL. In other words, a lower core to skin temperature gradient measured in the heat could be responsible for the diminution of the performance [7]. The highest speed over any 10% interval was therefore recorded in the last 10% for all three conditions, indicating that the participants had the ability to voluntarily activate skeletal muscles when cycling at maximum speed in the trials. With regard to the proposition that the CNS limits exercise-induced hyperthermia by reducing the power output of exercise, we can hypothesize that decreased motor control (at a cortical and/or spinal level) early in HUMID and DRY (at the 15th km) adjusted the motor unit recruitment. Interestingly, Trec was not different between DRY and NEUTRAL for the first 15 km or between HUMID and NEUTRAL for the first 10 km. However, %iEMG was diminished, even though the participants’ core temperatures did not reach critical values. Additionally, the assumption that a critical core temperature would be reached, leading to exhaustion [2,20], was not corroborated by our study, because the participants finished the time trials without a critically high Trec (39.2 ± 0.5 °C in HUMID, 39.1 ± 0.5 °C in DRY and 38.5 ± 0.4 °C in NEUTRAL) and showed no evidence of heat illness at the end of the trials. Furthermore, when participants experience dehydration under heat stress in hot/dry climate, this combination can lead to a reduction in skeletal muscle blood flow [21]. In hot/humid climate, sweating does not result in heat loss and athletes performing aerobic exercise will become increasingly dehydrated [22], with impaired endurance performance [23]. A recommendation to ingest 600 mL before arriving at the laboratory was given to the participants to prevent dehydration. In addition, they were allowed to drink ad libitum which has been demonstrated to assure an optimal rate of fluid absorption in the laboratory and in ecological experiments [24,25]. In the present laboratory study, the rate of BML was significantly higher in HUMID than in NEUTRAL). Yet, current studies completed in ecological conditions have suggested that BML is not directly linked to a decreased in aerobic performance [26]. Similar BMLs in hot/humid environment have been described for a 27-km trail running race, i.e., a decrease of 3.9 ± 1.1% [10]. Likewise, the authors demonstrated in a half-Ironman triathlon that athletes did not conserve their body mass within the recommended range of 2–3% [9], but this did not indicate that the participants were hypohydrated [27], even though urine osmolality was significantly augmented after the race compared with immediately before. Indeed, it has been demonstrated in well-trained unacclimatized male runners that fluid ingestion failed to provide any ergogenic benefit in attenuating thermoregulatory and circulatory stress during exercise not only in warm dry conditions, but also in warm humid environment [28]. We observed a decreased BML of 2.6 ± 0.5 kg·h^−1^ (equivalent to 2.7 ± 0.5 % of BMLs) in HUMID conditions. This result indicates that although sweat evaporation was unproductive, the sudation did not stop or diminish compared to DRY, even in acclimated participants, in accordance with previous observations [29].

Authors have reported that an increased Trec is correlated with RPE during a dynamic exercise [4,30]. Trec is usually considered to have a greater impact than Tskin on the impairment of aerobic performance [31]. Indeed, a 1 °C-variation in Trec provides from ~70% to 90% of the circulatory response at the skin level and rate of sweating, in the thermoregulatory mechanisms [32]. Nevertheless, during exercise in the heat, an elevation in the skin temperature will not only trigger the thermoregulatory mechanism, but might also initiate afferent signals from thermo-receptors in the cutaneous tissues, which could be responsible for thermal perception in the control of human thermoregulatory behavior. A correlation was shown between high skin temperature and thermal discomfort, and this could play an important role in the selection of the intensity of a prolonged self-paced exercise [33]. In our study, we found that mean Tskin was higher in HUMID compared with DRY at 15 and 20 km and throughout the HUMID trial compared with NEUTRAL. A rise in Tskin could thus generate afferent feedback that reduces the CNS recruitment of skeletal muscle. This would explain the decrease in %iEMG in HUMID at 11, 15 and 19 km compared with NEUTRAL, and at 15 and 19 km compared with DRY. It has in fact been suggested that the CNS combines several afferent signals from different systems involving respiration, heart, muscles and thermoreceptors, and that it regulates motor command in order to defend organ integrity during exercise [17,34]. We speculate that this concept of a “central governor” could be generalized to heat, given that working muscles generate heat, which is then enhanced by the environmental strain of a hot/humid climate.

## 5. Practical Applications

The present study demonstrated that although a tropical climate imposes high limitations in terms of thermoregulation, these constraints are managed. Performance is reduced but a critical core temperature is not reached and physical activity continues. The most important parameter for managing performance seems to be the skin temperature. Therefore, methods that decrease it or mimic cold sensations (i.e., such as cold or menthol vaporization on the skin during exercise) would certainly help athletes to perform better in tropical conditions. This idea is supported by our observations in moderately trained athletes involved in running/cycling activities [35,36].

We acknowledge certain limitations with this study which should be addressed in the future. First, the testing was performed on participants’ bicycle to favor their comfort. This allowed the speed measurement but limited the access to an exact power output. Second, the sample size was relatively small (n = 10), and statistical power could have been lacking to evidence sharper specific physiological responses in tropical climate, as compared with hot and dry climate. Future research on this topic will need to robustly examine the possible mechanisms underlying muscular fatigue in hot and dry but also in tropical environments. In particular, more studies are needed to explore the thermoregulation and performance in women in such environments. It could be verified if women would be less negatively impacted than men because of their different thermoregulation mechanisms.

## 6. Conclusions

We found that %iEMG activity significantly decreased during exercise only in the HUMID condition and this happened well before rectal the temperature reached 40 °C. We hypothesize that the participants adjusted the intensity of exercise by a proactive mechanism before the failure of thermoregulation in order to avoid hyperthermia. We do not know the mechanisms by which the brain can control muscle recruitment, but it seems that core/skin temperature, as previously discussed, is an indicator of possible thermoregulation, acting as a warning to limit the increase in central temperature.

## Data Availability

Data may be obtained from the corresponding author upon request.
